# Overall Quality of Life Assessment in the Patients Undergoing External Beam Radiation in Outpatient Radiation Oncology Department

**Published:** 2015-07-01

**Authors:** Rashmi Koul, Richard Tse, Erwin Karreman, Arbind Dubey, Patricia Tai

**Affiliations:** 1 Radiation Oncologist, Department of Radiation Oncology, Allan Blair Cancer Center, 4101 Dewdney Ave, Regina, SK, Canada S4T7T1; 2 Regina Qu’Appelle Health Region, Regina, SK, Canada; 3 University of Saskatchewan, Saskatoon, Canada

**Keywords:** Radiation, Oncology, Quality

## Abstract

**Background:** The impact of treatment on cancer patients’ quality of life (QoL) has been the focus of a variety of longitudinal studies in English literature for past decade. The measurement of patient-reported outcomes which includes health-related quality of life is a new important initiative which has emerged and grown over the past three decades. Following the development of reliable and valid self-reported questionnaires, health-related quality of life has been assessed in tens of thousands of patients and a wide variety of cancers. With growing information, feedback and experience, the quality of the health-related QOL studies has improved a lot. We expect in near future more methodologically robust studies will be done in a scientific way to answer unanswered questions.

**Methods:** As part of a Dean's summer project, a survey was undertaken to facilitate a more complete description of the quality of life experience in patients with histological diagnosis of cancer undergoing external beam radiation as an outpatient at Allan Blair Cancer Center, Regina, Canada. The questionnaires had two major components: depression and global QOL. The depression was measured by the Zung Self-Rating Depression Scale which is a short self-administered survey to quantify the depression status of a patient.

**Results:** Overall, only the equation associated with the outcome of QoL - Physical well-being was significant. That data indicated that only the variable of age was a significant predictor. A positive relationship was present indicating higher levels of depression when patients received chemotherapy or narcotics. Breast cancer patients were less depressed than lung cancer patients.

**Conclusion:** Cancer and its related treatment is an important health issue influencing QoL. The study has revealed that the use of chemotherapy and narcotics has a significant impact on the quality of life (QoL).

## Introduction

 Quality of Life (QoL) assessment in cancer clinical trials provides a more accurate evaluation of the well-being of individuals or groups of patients and of the benefits and side-effects that may result from medical intervention.^1^ Under normal circumstances, most of us would probably define QoL as companionship with family and friends, rewarding work (paid or volunteer), the knowledge that we make a difference in the lives of others, the freedom to pursue a multitude of interests and the joy of learning something new, but under the abnormal circumstance of receiving a cancer diagnosis and undergoing aggressive cancer treatment, these sources of satisfaction and self-esteem can be severely compromised.^2^ QoL diminishes very quickly when one is fearful, fatigued, in pain, enduring therapeutic side effects or contemplating the possibility of treatment failure and death. Therefore, our first task in dealing with cancer is to regain some sort of equilibrium which will include mental, physical and spiritual aspects of that individual by addressing these very real issues and creating a support system tailored to our patient and their needs.^3^

## MATERIAL AND METHODS

 As part of a Dean’s summer project for medical students, a survey was undertaken to facilitate a more complete description of the QoL experience in patients with histological diagnosis of cancer undergoing external beam radiation as an outpatient at Allan Blair Cancer Center, Regina, Canada. The enrollment was entirely on voluntary basis. Initial contact was made during the middle of external beam radiation and questionnaires were filled by the patients. Patients had right to refuse or even withdraw at any time. They were allowed to refrain to answer any question as per their comfort level. The questionnaires took 10-15 minutes to complete. 

The questionnaires had two major components: depression and global quality of life (QoL). The depression was measured by the Zung Self-Rating Depression Scale which is a short self-administered survey to quantify the depression status of a patient.^4^ There are 20 items on the scale that rate the four common characteristics of depression: the pervasive effect, the physiological equivalents, other disturbances, and psychomotor activities. There are ten positively worded and ten negatively worded questions. Each question is scored on a scale of 1-4 (a little of the time, some of the time, good part of the time, most of the time). The scores range from 25-100: 25-49 normal range, 50-59 mildly depressed, 60-69 moderately depressed, 70 and above severely depressed. For QoL we administered FACT-G questionnaire.^5^ As we all know, QoL research provides patients and health-care providers with vital information about the impact that disease and its treatment has on physical, functional, social and emotional well-being. QOL outcomes are also being recognized as important prognostic variables, which help to predict which patients are most likely to benefit from treatment. The FACT-G has good psychometric properties supporting its broad generalizability. *FACT-F *questionnaire has been used to evaluate symptoms resulting from cancer treatments such as chemotherapy^6^ and radiotherapy,^7^ as well as the efficiency, dosage and security of medicines for chemotherapy-induced anemia^8^^,^^9^ in interventions involving exercises in patients with cancer and fatigue^10^ in complementary cancer therapy^11^ and in nursing interventions.^12^

*FACT-F *consists of a questionnaire with a total of 40 items, which consists of 27 items, *the* *Functional Assessment of Cancer Therapy-General (FACT-G)*, evaluating global quality of life, and 13 specific items relating to fatigue.^13^ *FACT-F *is part of the measure system, *the Functional Assessment of Chronic Illness Therapy *(FACIT), which comprises a collection of health-related QoL questionnaires**. **These questionnaires were developed to be applied to patients with chronic diseases.^14^ All FACIT questionnaires were submitted to a standardized development with valid methodology that passes through five phases: (1) generation of the item, (2) revision and reduction of the item, (3) construction of the scale, (4) initial evaluation and (5) additional evaluation for the whole system measure. They are available in 45 languages, allowing the comparison of different populations, using a rigorous methodology of translation and back-translation.^15^ Use of this scale with the addition of the Zung Self-Rating Depression Scale gives a well-rounded view of the various aspects of QoL from the patient's perspective.^16^

## Results

 Means and standard deviations for the four quality of life measures and the depression measures are reported in Table 1. This table also includes the bivariate correlations (alphas are reported on the diagonal). The results of the five initial regressions used to evaluate the influence of the potential confounding variables on the five outcome variables are reported in Table 2. Overall, only the equation associated with the outcome of QoL - Physical well-being was significant. 

**Table1:** Means, standard deviations, and correlations for the included quality of life variables and depression (alpha values in the diagonal)

**N**=**100**	**M (SD)** **	**1**	**2**	**3**	**4**	**5**
**1. Quality of life - Physical well-being**	3.91 (0.82)	.88				
**2. Quality of life - Social/family well-being**	4.31 (0.72)	.11	.70			
**3. Quality of life - Emotional well-being**	4.16 (0.72)	.33*	.25*	.70		
**4. Quality of life - Functional well-being**	3.73 (0.92)	.52*	.27*	.29*	.88	
**5. Depression**	1.85 (0.42)	-.63*	-.26*	-.38*	-.69*	.73

* **Note** ***.*** **p** < .05.

** Variables 1, 2, 3, and 4 scored on 5-point scale (1-5); variable 5 is 4 point measures (1-4) Higher scores reflecting greater levels of the measured variable

**Table 2:** Results of initial regression equations exploring the impact of potential confounding variables on outcome variables

	**Quality of life** ** - Physical ** **well-being**	**Quality of life – ** **Social/family ** **well-being**	**Quality of life – ** **Emotional well-** **being**	**Quality of life – ** **Functional well-** **being**	**Depression**
**Overall ** ***R*** ^2^ ***F***_(2,92)_	.09 4.3*	.02 0.9	.01 0.2	.02 0.8	.01 0.3
**Std. Beta Weights**					
**Age**	.28*	-.10	-.05	.13	-.02
**Sex**	.04	-.08	.06	-.03	.08

* ***Note.*** **p**<.05

indicated that only the variable of age was a significant predictor. This variable was retained and entered in the first block of the hierarchical regressions when the outcome variable was QoL - Physical well-being. Table 3 reports the results of the subsequent regressions. Overall regression equations were significant (*p* <.05), except for quality of life - social/family well-being. *F* values ranged from 1.1 to 8.3. Whether patients received chemotherapy or narcotics were the most important predictors of quality of life. Patients who received chemotherapy or narcotics scored lower on these quality of life measures. Both predictors were significant for at least half of the quality of life measures with all significant standardized betas indicating a large effect (<.25, Keith, 1999). Also, both measures were the only significant predictors of depression and both effects were considered large. A positive relationship was present, indicating higher levels of depression when patients received chemotherapy or narcotics. Patients who experienced cancer in the past or treated with radiation therapy had a significant positive relationship with emotional well-being subscale of the quality of life measure. It showed that such patients had higher scores on emotional well-being. 

**Table 3:** Results of regression equations: Overall R2 and standardized beta coefficients for predictor variable

	**Week of radiation ** **(β)**	**Past cancer ** **(β)**	**Chemo therapy** **(β)**	**Hormones ** **(β)**	**Narcotics** **(β)**
**Quality of life - Physical well-being** a **(*R***^2^ = .43*; Δ*R*^2^, Block 2 = .31*)	-.04	.06	-.26*	.15	-.49*
**Quality of life - Social/family well-being** **(** ***R*** ^2^ ** = .07)**	.15	.21	-.15	.01	.11
**Quality of life - Emotional well-being** **(** ***R*** ^2^ ** = .15** * **)**	.30*	.22*	-.33*	.00	-.11
**Quality of life - Functional well-being** **(** ***R*** ^2^ ** = .26** * **)**	.15	.13	-.27*	.02	-.37*
**Depression (** ***R*** ^2^ ** = .31** * **)**	-.17	-.11	.30*	-.20	.32*

* ***Note*** *.* *p *< .05;

a **–** standardized beta taken from final step

None of the other regressions with these two predictors was significant. Reception of hormones was not a significant predictor of any of the outcome variables. In addition, two groups of patients with different types of cancer (lung cancer, n = 11 and breast cancer, n = 23) were compared on the variables of interest. Results indicated that breast cancer patients rated quality of life - functional well-being significantly higher than lung cancer patients *t *(31) = -2.82, *p* >0.05. This represented a medium to large-sized effect (*r* = 0.45). Breast cancer patients also scored significantly lower on the measure of depression than lung cancer patients *t *(31) = 4.46, *p* >0 .05, which represented a large-sized effect (*r* =0.62).

## Discussion

 In literature there are several instruments available to measure different domains that can affect quality of life.^17^ *FACT-G *specifically was developed and validated to measure the quality of life in adult patients with cancer and is now in its 4^th^ version.^18^ Its 27 items contemplate four domains: physical well-being, social/family well-being, emotional well-being and functional well-being. It is considered appropriate for patients with any type of cancer. *FACT-G *was conceived originally in English and submitted to the translation process into Portuguese, which included two translations, a reconciliation translation, a back-translation of the reconciled version and four independent revisions by bilingual experts.^19^ It was pre-tested in 19 cancer patients in Portugal and 30 in Brazil. One of the stages for validating a questionnaire is the test - retest of the version translated to Portuguese. In this study the instrument *FACT-F *was applied to 85 patients with different types of cancer. The participants of this research had mainly stages III and IV cancers and more than 50% of patients presented with advanced disease at diagnosis. Sixty-three per cent of individuals were interviewed, in which questionnaire items were read and filled out by the interviewer. Because the elderly represented the majority of patients who had low educational levels.^20^ In another study, the evolution of health-related quality of life (HRQoL) in a cohort of breast cancer patients over 1 year after surgery was evaluated and the predictive ability of HRQoL measurement instruments was analyzed. A total of 364 women participated in the study. EuroQoL visual analogue scale (VAS) scores improved (1 month vs. 1 year: 70 vs. 80; p<0.0001), however, the EuroQoL score showed no significant change (0.81 vs. 0.83; p=0.1323).^21^ HRQoL data after treatment for early-stage Hodgkin's lymphoma have shown that patients experience strain and limitations in all subdomains apart from cognitive functioning (QLQ-C30) and also reduced motivation (MFI-20). Differences in HRQoL improvement with time were linked to age and sex, but not type of treatment. Fatigue status at the end of treatment seems to predict subsequent HRQoL.^22^

Another popular tool in literature is Edmonton symptom assessment scale (ESAS). The ESAS was designed for palliative patients. Nine VASs evaluate symptoms (activity, anxiety, appetite, depression, drowsiness, pain, nausea, shortness of breath and well-being); the scores are summated to a distress score. It is valid with internal consistency and test-retest reliability.^23^ Routine use of ESAS on admission to a palliative care unit has shown significant underassessment and documentation of symptoms, especially inactivity, impaired well-being, and anxiety.^24^

Overall, we feel that today the oncological management is patient centric so, much of the work of HRQoL development has centered on its use during the patient visit. Patients are no longer regarded as passive recipients of services. They should be able to determine how much information they want and how much they want to participate in decision making and self-care. Use of patient-reported HRQOL information implies shared decision making because it requires that both patient and clinician be knowledgeable about the effects of disease and treatment. The clinical information should not be viewed simply as an archive of stored medical data documenting past events and findings. Rather, clinical information is the flow of knowledge to whoever needs it in caring for a patient, whether face-to-face or by various forms of electronic communication. With internet based applications, medical records can be held physically or digitally in a variety of locations to be accessed in whole or in part by the patient or anyone to whom he or she grants permission, as has been pioneered in Boston’s Care Group.^25^

## CONCLUSION

 Health-related QoL has become a more accurate predictor of survival than some other clinical parameters, such as performance status in literature. If health care professionals can identify patients who are not doing well, they will be able to intervene and improve not only their sense of well-being but also their survival by an appropriate intervention. Importantly, the data which patients provide will give us information about long-term needs or factors that affect their quality of life. However, more research is required to identify the interventions which will yield positive impact on the oncology patients.
